# Geographic Distribution of Habitat, Development, and Population Growth Rates of the Asian Citrus Psyllid, *Diaphorina citri*, in Mexico

**DOI:** 10.1673/031.013.11401

**Published:** 2013-10-26

**Authors:** José López-Collado, J. Isabel López-Arroyo, Pedro L. Robles-García, Magdalena Márquez-Santos

**Affiliations:** 1 Colegio de Postgraduados, Campus Veracruz, Km 88.5, carretera federal Xalapa-Veracruz, C.P. 91690; 2 Instituto Nacional de Investigaciones Forestales, Agrícolas y Pecuarias, Campo Experimental General Teran, Km 3.1 Carretera Montemorelos-China Ex hacienda Las Anacuas, C.P. 67400; 3 Dirección General de Sanidad Vegetal, Depto. Campañas de Prioridad Nacional, Guillermo Pérez Valenzuela 127, Col. del Carmen Coyoacán, México D.F. C.P. 04100

**Keywords:** citrus pest, huanglongbing, niche estimation, species distribution modeling

## Abstract

The Asian citrus psyllid, *Diaphorina citri* Kuwayama (Hemiptera: Psyllidae), is an introduced pest in Mexico and a vector of huanglongbing, a lethal citrus disease. Estimations of the habitat distribution and population growth rates of *D. citri* are required to establish regional and areawide management strategies and can be used as a pest risk analysis tools. In this study, the habitat distribution of *D. citri* in Mexico was computed with MaxEnt, an inductive, machine-learning program that uses bioclimatic layers and point location data. Geographic distributions of development and population growth rates were determined by fitting a temperature-dependent, nonlinear model and projecting the rates over the target area, using the annual mean temperature as the predictor variable. The results showed that the most suitable regions for habitat of *D. citri* comprise the Gulf of Mexico states, Yucatán Peninsula, and areas scattered throughout the Pacific coastal states. Less suitable areas occurred in northern and central states. The most important predictor variables were related to temperature. Development and growth rates had a distribution wider than habitat, reaching some of the northern states of México. Habitat, development, and population growth rates were correlated to each other and with the citrus producing area. These relationships indicated that citrus producing states are within the most suitable regions for the occurrence, development, and population growth of *D. citri*, therefore increasing the risk of huanglongbing dispersion.

## Resumen

El psílido asiático de los cítricos, *Diaphorina citri* Kuwayama (Hemiptera: Psyllidae) es una plaga introducida en México, y es vector del huanglongbing, una enfermedad letal de los cítricos. La estimación de la distribución del hábitat y tasas de crecimiento poblacional de *D. citri* se requieren para establecer una estrategia de manejo a nivel regional y como herramienta de análisis de riesgo. En este estudio, la distribución del hábitat de *D. citri* en México se calculó con MaxEnt, un programa inductivo, de aprendizaje-máquina que emplea capas bioclimáticas y datos puntuales de localización. También se determinaron las distribuciones geográficas de las tasas de desarrollo y crecimiento poblacional por el ajuste de un modelo no-lineal, dependiente de la temperatura y proyectando las tasas sobre el área objetivo usando la temperatura media anual como variable predictora. Los resultados mostraron que las regiones mas apropiadas para el hábitat de *D. citri* comprenden los estados del Golfo de México, Península de Yucatán y áreas dispersas de los estados costeros del Pacífico. Áreas menos apropiadas ocurren en los estados del centro y norte. Las variables predictoras más importantes estuvieron relacionadas con la temperatura. Las tasas de desarrollo y crecimiento poblacional presentaron una distribución más amplia que el hábitat, alcanzando algunos de los estados del norte. El hábitat y las tasas de desarrollo y crecimiento poblacional estuvieron correlacionadas entre sí y con la superficie plantada con cítricos. Estas relaciones sugieren que los estados productores de cítricos se encuentran dentro de las áreas mas apropiadas para la ocurrencia, desarrollo y crecimiento poblacional de *D. citri*, por tanto incrementando el riesgo de dispersión del huanglongbing.

## Introduction

The Asian citrus psyllid, *Diaphorina citri* Kuwayama (Hemiptera: Psyllidae) is an important pest of commercial citrus worldwide. It has a variable life cycle, and egg to adult development is temperature dependent (Liu and Tsai 2000), as is the population growth rate, as measured by the intrinsic rate of increase (rm) (Nava et al. 2010). *Diaphorina citri* feeds on shoots, leaves, and buds of citrus plants (Yang et al. 2006). Also, it vectors the pathogen associated with huanglongbing, a lethal citrus disease (da Graça and Korsten 2004; Bové, 2006). The pest has a worldwide distribution across tropical and subtropical countries (Yang et al. 2006). In Mexico, *D. citri* was detected in 2002 (López-Arroyo et al. 2009) and it has dispersed throughout the country. Its presence has been reported in all of the citrus producing states (López-Arroyo et al. 2009).

Huanglongbing is a bacterial disease caused by the phloem-limited a-proteobacteria *Candidatus* Liberibacter spp (Rhizobiales: Rhizobiaceae). Three species of *Candidatus* have been determined: *Ca.* L. asiaticus, *Ca.* L. africanus, and *Ca.* L. americanus (Bové 2006). Huanglongbing threatens the citrus industry because it causes the death of the trees and is difficult to control. The disease is considered a serious factor affecting the production of commercial citrus orchards, it has no cure, and only preventive measures can deter the spread of the disease once it has been established. Huanglongbing occurs in countries in Africa, Asia, and America (Bassanezi et al. 2010). It is present in Brazil, U.S.A., Central America, Caribbean Islands, and Mexico (National Research Council 2010). In Brazil, huanglongbing caused the removal of thousands of trees, and producers have allocated economic resources to reduce damage and consequently decrease the dispersion (Bassanezi et al. 2010). In Mexico, the disease was first detected in Yucatan in 2009, and after that it appeared in some municipalities across the Pacific coastal states (SENASICA 2011). Regulatory control measures have been undertaken to control the disease, and a nationwide alert campaign has been launched, mainly in citrus producing states (Mayorga 2010). Mexico ranks among the five top citrus producing countries worldwide (FAO 2008), so the occurrence of *D. citri* and huanglongbing is a serious threat to the Mexican citrus industry (Salcedo et al. 2010).

The geographic distribution of introduced pests is an important component for risk analysis and making decisions on control methods. Species distribution modeling is a key tool to study the capacity of a species to occur in a given region (Elith and Leathwick 2009). Estimates are based on deductive, inductive, and hybrid methods. Deductive methods compute species distribution based on climatic requirements and do not require presence data. Inductive methods rely on actual sampling points, thus providing an estimate based on presence of the pest. Hybrid methods combine both (Venette et al. 2010). MaxEnt is an inductive, machine-learning program that computes the habitat or niche distribution for a given organism (Phillips et al. 2006) and has performed well when compared to other methods (Ortega-Huerta and Peterson 2008). However, for a large sample size, boosted decision trees performed slightly better than MaxEnt, but they did not differ significantly from each other (Wisz et al. 2008). Like other distribution models, MaxEnt uses environmental layers and presence-only data to predict habitat distribution for a given organism (Phillips et al. 2006); point occurrence data are most commonly generated, as compared to presence-absence data (Tsoar et al. 2007; Elith et al. 2011).

The habitat distribution gives a view of where a species could establish, but development and population growth rates are biophysical processes that provide additional information on how insect populations respond to abiotic factors (Buckley et al. 2010). Most studies have focused on studying the effect of temperature on these rates, because insects are poikilothermic organisms (Wagner et al. 1984). The development and population growth of *D. citri* has been measured on different citrus species, including the orange jasmine, *Murraya paniculata*, and degree-days models have been computed to describe development (Nava et al. 2010). However, the development of insects is intrinsically non-linear (Wagner et al. 1984), and, to account for the variability in temperatures occurring at country level, this non-linearity should be included. Several nonlinear models have been proposed and used to represent temperature-dependent variables, which range from three- to six-parameter models (Wagner et al. 1984). Briere 1 model has been compared to more complex models with similar performance; nonetheless, it uses only three parameters, allowing it to handle small datasets (Shi and Ge 2010), and it has been applied to model aphid development, among other organisms (Jalali et al. 2010). In addition to habitat prediction, an estimate of the distribution of potential growth and development may shed light on the biological capacity of *D. citri* to thrive in the different agroclimatic regions in Mexico. Given that huanglongbing is already present in Mexico, the objective of this study was to know the potential distribution of habitat, development, and population growth rates of *D*. *citri* in this country.

## Materials and Methods

### Habitat distribution

Habitat distribution of *D. citri* was estimated with MaxEnt version 3.3.3 (Phillips et al. 2006). MaxEnt generates a variable that measure the probability to occur in a given region and is considered as the realized niche (Phillips et al. 2006); probability ranges from 0 (least likely) to 1 (most likely) to occur. The model requires two types of data: species point location data and bioclimatic layers. *Diaphorina citri* point location data were collected and geo-referenced by personnel of the Mexican Plant Health State Committee during 2008 and 2009 across most of the citrus producing states as a result of a nationwide huanglongbing detection effort (Robles-García and Delgadillo-Villanueva 2008). Bioclimatic data were retrieved from Atlas Digital de Mexico (Fernandez-Eguiarte et al. 2010), comprising 19 raster layers derived from temperature and precipitation and representing extreme values, seasonality, and annual trends (Table 1). These layers were curated from the WorldClim database (www.worldclim.org), were derived from historic global source data ranging from 1950 to 2000, and had a spatial resolution of 30″ or about 0.86 km^2^ at the Equator; these layers were generated by interpolation with a thinplate smoothing spline algorithm (Hijmans et al. 2005). The fitness of the model was measured by reserving 30% of sampling points for testing purposes (test data) and computing the area under the curve (AUC) to compare the predictive power of the model when using the test data against the training data (Phillips and Dudík 2008). AUC values less than 0.7 indicate a low performance model, values between 0.7 and 0.9 indicate a useful model, and values higher than 0.9 indicate a very good model (Baldwin 2009). The importance of the bioclimatic variables was estimated with jackknife where the relative contribution of each variable was evaluated either as its single contribution or by removing it from the predictor variable set (Phillips et al. 2006). For the rest of the model parameters, the default values were used, as recommended by Phillips and Dudík (2008). The citrus growing area was not used as a bioclimatic layer due to its low resolution; instead, a regression analysis at state level was performed and described later.

**Table 1.**
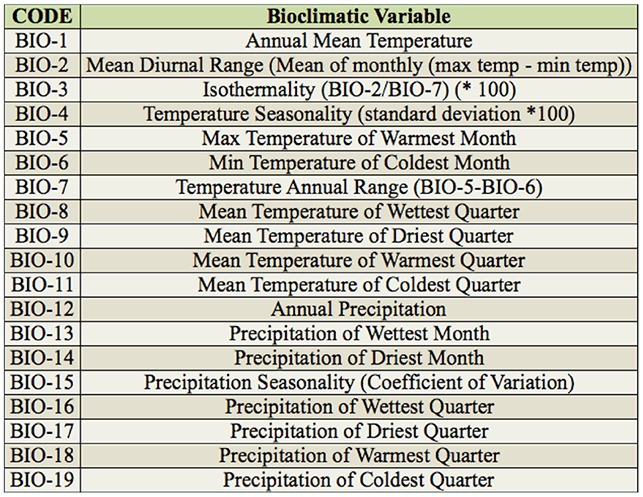
Bioclimatic variables used to fit the Maxent model.

### Development and population growth rate Distributions

The geographical potential for development rate, which is the reciprocal of development time (1/d) and population growth rate (rm), was computed in a different way as for habitat and applied a biophysical model that describes these indices as a function of temperature. A two-step approach was used: first, the parameters of the Briere 1 model (Briere et al. 1999) were estimated as a function of temperature using the NLIN procedure with SAS v. 9.1 (SAS Institute 2004). The model is:

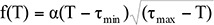
where f(T) is either development rate (1/d) or the intrinsic rate of increase (rm) as a function of temperature (T). The model parameters are: α, an empirical constant and τ_min_ and τ_max_, which represent the minimum and maximum threshold temperatures; T is the constant temperature at which individuals are kept to measure their development.

Development rate data, from egg to adult, were used to fit the model and were obtained from Nava et al. (2010). For the intrinsic rate of increase, data published by Liu and Tsai (2000) were analyzed. In the second step, once the model parameters were estimated (α, τmin, and τmax), the geographic distribution of 1/d and rm was calculated by using the average annual temperature raster layer for the whole country as the predictor variable T (Fernandez-Eguiarte et al. 2010) and was used as input to the Briere 1 model to obtain the corresponding response f(T), that is, T is the raster layer containing the average annual temperature and α, τmin, and τmax substituted by their nonlinear estimates. These map algebra operations were done with Octave version 3.2 (Eaton et al. 2008). Finally, to determine the similarity among the three suitability indices (probability, 1/d, and rm) and its relationship with the citrus growing area (ha) for year 2009 (SIAP 2010), three linear models of the form Y = β_0_ + β_1_ X were estimated at state level; therefore, average distribution values were computed at this level. For each distribution, the state area was clipped with the corresponding state polygon, and then the average value was computed. These operations were performed with SAGA version 2.0.7 (SAGA 2011). For the relationship between probability and the citrus growing area, mean values were transformed to natural logarithms (ln). The model parameters β_0_ and β_1_ were estimated by regression analysis using PROC REG in SAS version 9.1 (SAS Institute 2004). All the maps were composed by overlaying the distribution raster layers with the political boundaries with ArcGis version 9.3.1 using a WGS84 projection (ESRI 2009); the state-level vector layer was obtained from INEGI (INEGI 2011).

## Results

### Habitat distribution of *Diaphorina citri*

**Figure 1.**
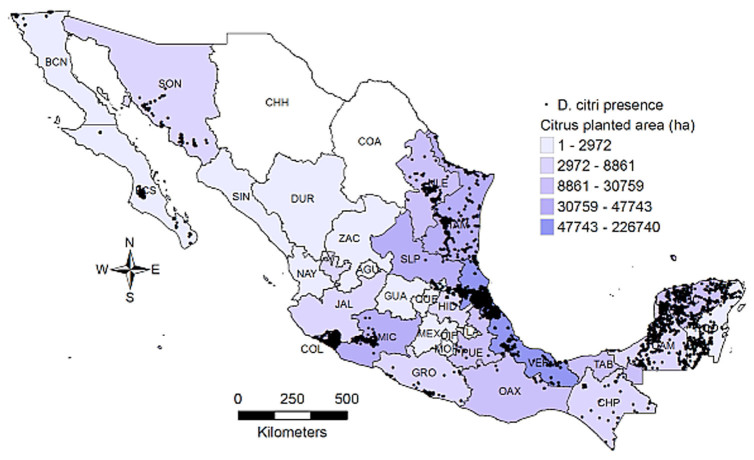
Sites with *Diaphorina citri* presence during 2008–2009, and citrus production area (ha) for the Mexican states during year 2009. High quality figures are available online.

**Figure 2.**
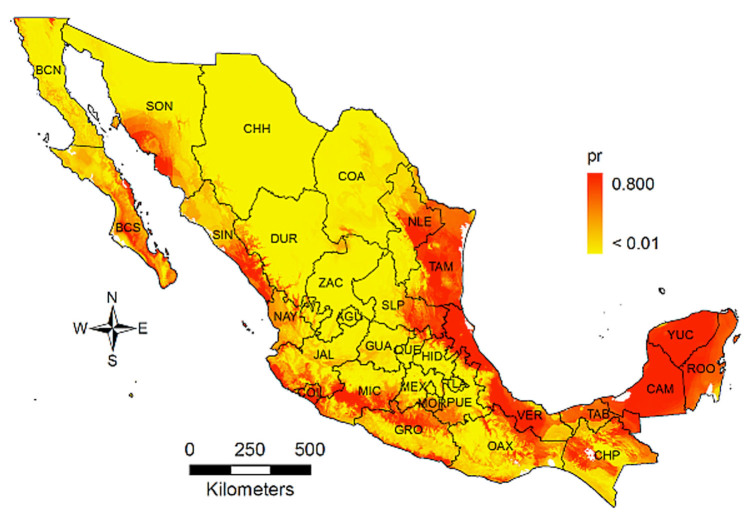
Probability (pr) of habitat distribution of *Diaphorina citri* estimated by MaxEnt. High quality figures are available online.

The presence of *D. citri* in Mexico was detected for most of the citrus producing states (Figure 1) and reflected the effort to sample the most important citrus growing areas, such as Colima, Michoacan, and Veracruz, and the potential huanglongbing entry points such as the northern border states (Baja California, Nuevo Leon, Tamaulipas) and the Yucatán peninsula. The estimated probability of habitat distribution of *D. citri* is shown in Figure 2. High values of presence occurred in parts of Baja California Sur, Sonora, Sinaloa, Colima, Michoacan, and Guerrero, along with the Gulf of Mexico states and the Yucatán peninsula. Furthermore, northern and central states like Chihuahua, Coahuila, Durango, Zacatecas, Aguascalientes, and Guanajuato had the lowest values. In general, *D. citri* had a high, uniform distribution in all of the Gulf of México states and was scattered throughout the Pacific states.

**Figure 3.**
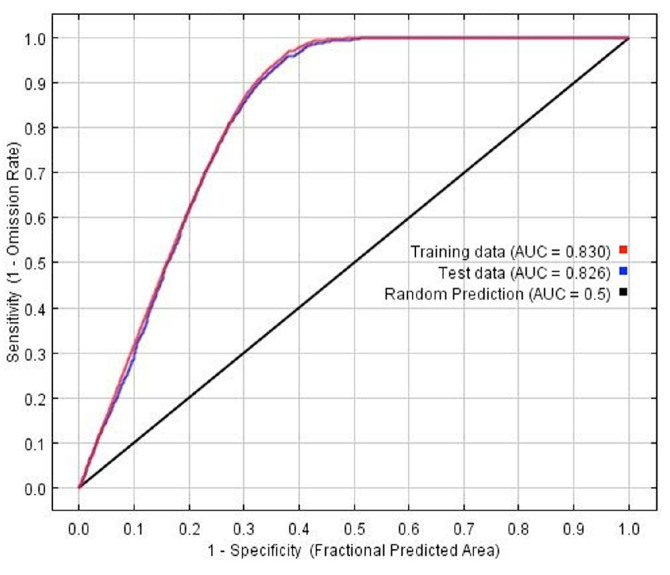
Area under the curve (AUC) for estimated *Diaphorina citri* distribution in México, as modeled by MaxEnt. High quality figures are available online.

**Figure 4.**
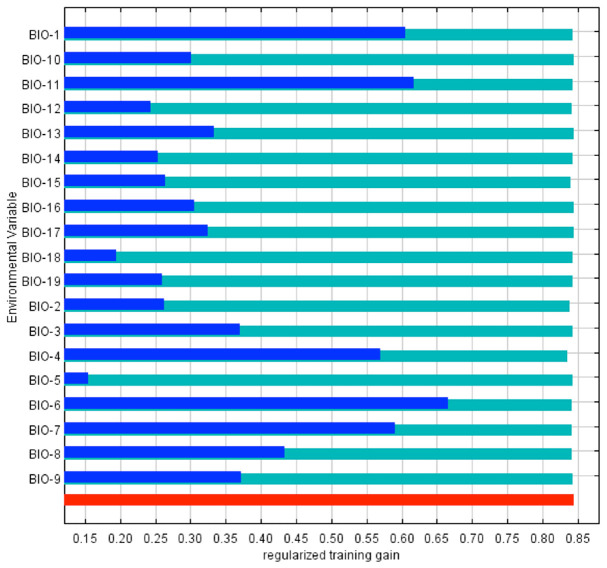
Jackknife test of variable importance for train data: individual variable contribution (blue bar), contribution when a given variable is excluded (green bar), whole set of variables (red bar). Variable names are listed in Table 1. High quality figures are available online.

**Figure 5.**
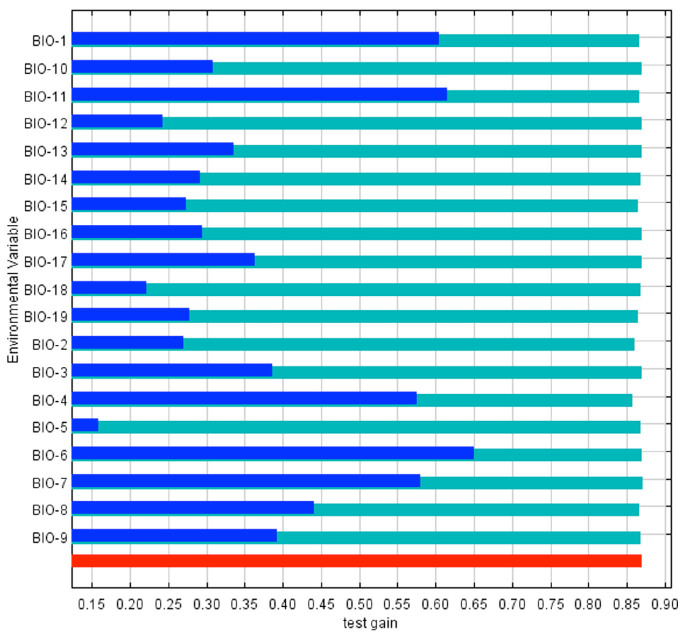
Jackknife test of variable importance for test data: individual variable contribution (blue bar), contribution when a given variable is excluded (green bar), whole set of variables (red bar). Variable names are listed in Table 1. High quality figures are available online.

The model fitness for the estimated *D. citri* distribution was measured with the AUC (Figure 3). The red line indicates the fit with the training data, and the blue line indicates the actual model performance with the independent test data. Both were similar in their predictive value, and model prediction is statistically different from a random one (*p* < 0.01, binomial test). AUC values over 0.7 are considered appropriate for predictive purposes (Phillips and Dudík 2008; Baldwin 2009); therefore, the model was deemed appropriate to show where *D*. *citri* can occur. MaxEnt uses several bioclimatic layers for predictive purposes; however, their relative importance differs among them. The contribution of the predictor variables to model fitting (train data) is shown in Figure 4. The five most important by their individual contribution (blue bars) were temperature-related, two refer to extreme cold (BIO-6, BIO-11), and the others to average (BIO-1) or fluctuating values (BIO-4, BIO-7); the less important variables were related to high temperatures and to precipitation. The same figure shows that when a variable is excluded from the model (green bars), it contains less valuable information than by itself, that is, the whole model is more resilient to variable exclusion. On the other side, Figure 5 shows variable contribution for the test data and has similar values as those for model fitting. Overall, the most important variables have critical information either to fit the model (train data) or to predict values (test data).

### Development and population growth rate Distributions

**Figure 6.**
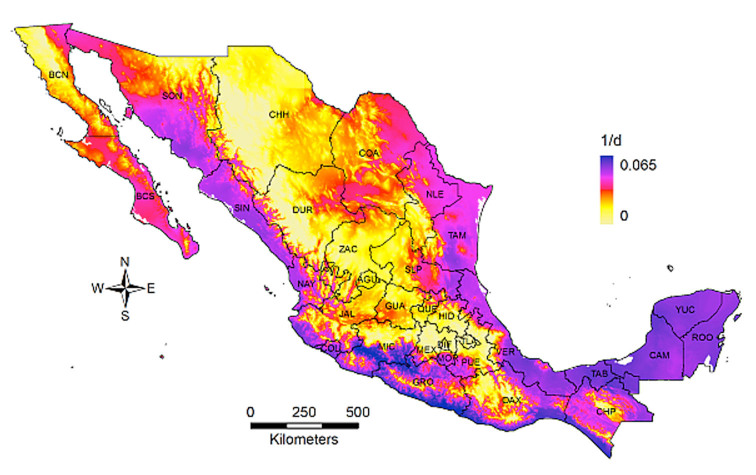
Distribution of development rate (1/d) of *Diaphorina citri* in México. High quality figures are available online.

The Briere 1 model was significant for development (*F*_3,3_ = 447.79; *p* < 0.01). The estimated parameters were α = 4.5E-5, τ_min_ = 12.6° C, and τ_max_ = 38.1° C. When this model was applied using the annual mean temperature raster layer T as the input, the development rate had a distribution wider than niche, presenting high values along most of the coastal states, the Yucatán peninsula, and Baja California Sur (Figure 6). This result indicates that *D. citri* had a high potential for development in most of the suitable locations. MaxEnt estimates were constrained by the presence-only records, while the computations of (1/d) were constrained only by the lower and upper threshold temperatures, thus the wider distribution.

**Figure 7.**
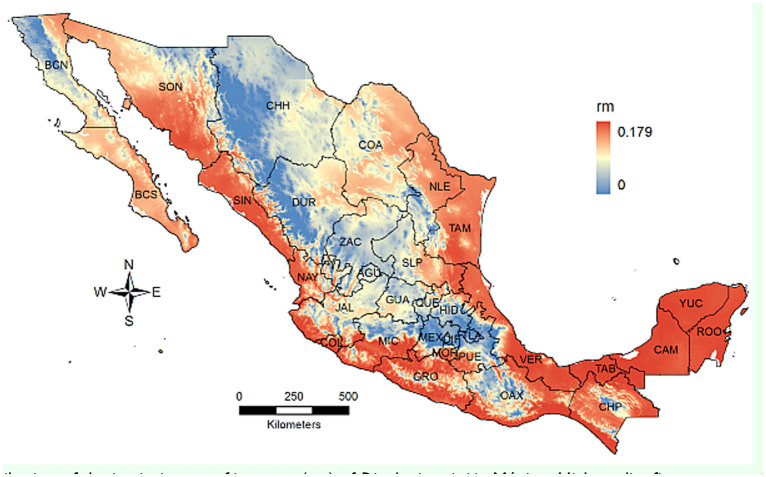
Distribution of the intrinsic rate of increase (rm) of *Diaphorina citri* in México. High quality figures are available online.

Regarding the potential of population growth, the parameters for the Briere 1 model were α = 2.3E-4, 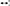
_min_ = 13.2° C and 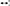
_max_ = 31.4° C, and the effect of temperature was significant (*F*_3,2_ = 61.83; *p* = 0.016). This model was solved over the annual mean temperature T, and the resultant geographic distribution of rm is shown in Figure 7. Like the distribution of development rate, the higher values occurred along the coastal states of the Pacific Ocean and the Gulf of México, including the Yucatán peninsula and Baja California Sur. Therefore, these regions presented a high potential for the population to grow.

**Figure 8.**
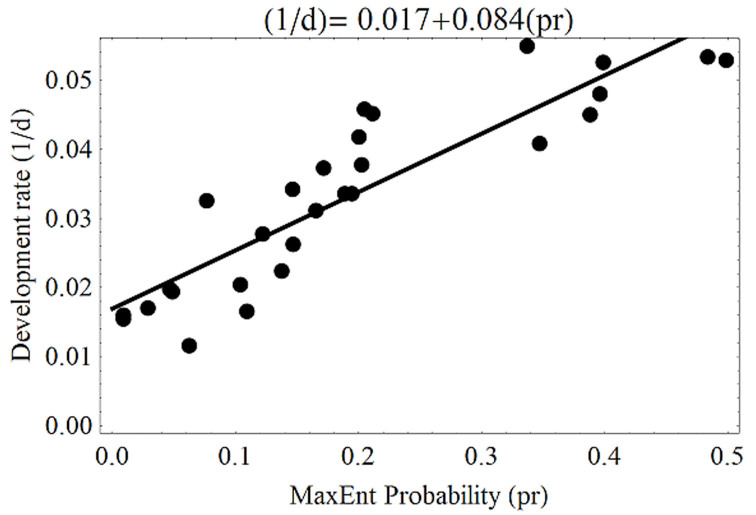
Relationship between habitat distribution probability values (pr) and development rate (1/d) at state level. High quality figures are available online.

**Figure 9.**
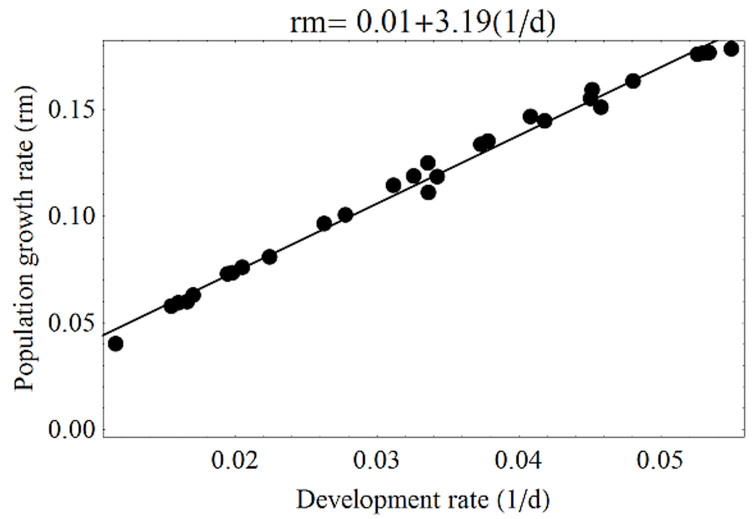
Relationship between development rate (1/d) and population growth rate (rm) at state level. High quality figures are available online.

**Figure 10.**
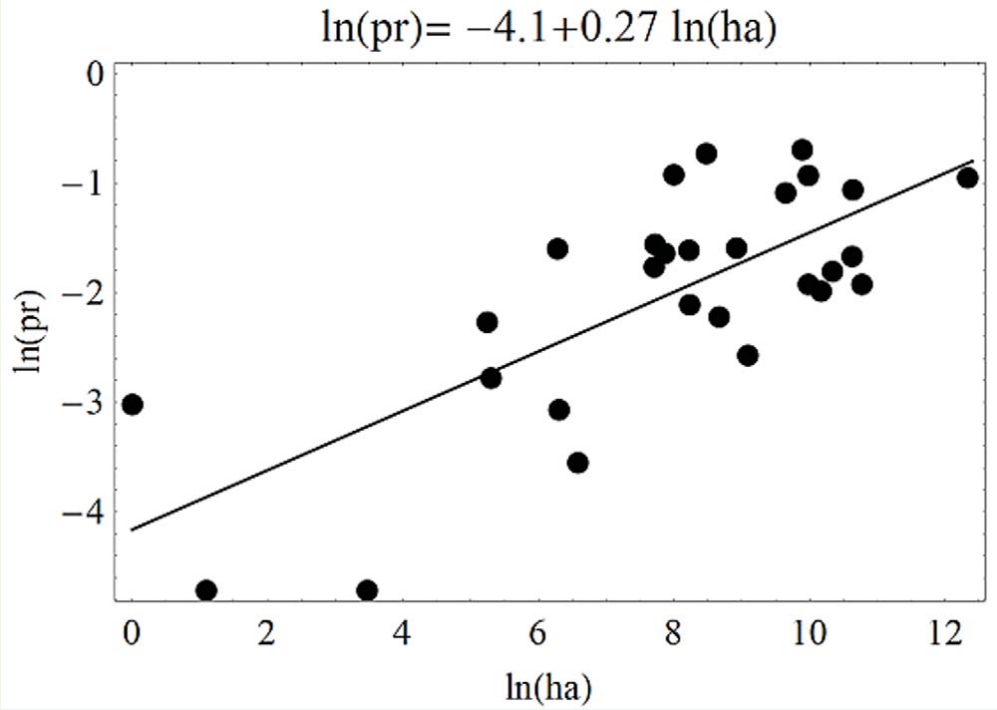
Relationship between citrus planted area ln(ha) and occurrence probability ln(pr) for citrus producing states. High quality figures are available online.

At the state level, the three indices were related. First, development rate and habitat had a significant linear relationship (*F*_1,26_ = 103.4, *p* < 0.01; *r*^2^ = 0.79) (Figure 8), hence the similarity in their distribution maps. Second, the relationship between population growth and development rates showed a closer similarity (*F*_1, 26_ = 2947.7, *p* < 0.01; *r*^2^ = 0.99) (Figure 9) because both are affected in a similar way by temperature. Third, the effect of citrus host on habitat was estimated by the relationship between habitat distribution and the citrus growing area, and this was significant (*F*_1, 26_ = 31.3, *p* < 0.01) (Figure 10). In this case, however, the trend was coarse, given the medium value of *r*^2^ (0.54). This model indicated that the larger the growing area, the higher the probability of pest presence.

## Discussion

### Habitat distribution of *Diaphorina citri*

*Diaphorina citri* was first detected in Mexico in 2002 and it has extended to most of the country (López-Arroyo et al. 2009). It is thought to be native from tropical and subtropical regions of Asia (Liu and Tsai 2000; Halbert and Manjunath 2004; Yang et al. 2006). The habitat distribution estimated with MaxEnt roughly agreed with the biogeographical region known as the neotropical zone (Morrone 2002), which ranges from Central America to central and southern Mexico, and was consistent with the distribution of other psyllid species, such as *Heteropsylla cubana* (Hodkinson 1989). However, some of these regions had low probability values, such as Guerrero, Oaxaca, and Chiapas. Host occurrence is an important factor affecting *D. citri* distribution but was not included in the MaxEnt analysis; in fact, Guerrero, Oaxaca, and Chiapas have small areas planted with citrus (Figure 1), thus the chance to find *D. citri* was reduced. On the contrary, northern states like Nuevo Leon and Tamaulipas, which are beyond the neotropical region, had high probability values and large areas planted with citrus (SIAP 2010). Therefore, the distribution of *D. citri* depends on the availability and distribution of citrus plants as well (Robles- García and Delgadillo-Villanueva 2008). This factor was accounted for by the relationship between probability values and the surface planted with citrus, as modeled by the linear model (Figure 7), and was significant, as expected.

Moreover, the main variables affecting probability distribution were related to temperature, from which the minimum temperature of coldest month seemed to be the most important; in consequence, it appears that presence was most affected by cold temperatures. These findings agree with those reported by Yang et al. (2006), who cite ambient temperature as the most important factor limiting the distribution of *D. citri*, and particularly average temperature of the coldest month, minimum temperature, and the duration of cold weather. Extreme temperatures have long been considered to affect plant distribution (Magarey et al. 2008), and, for insects, non-linear development models explicitly account for minimum and maximum temperatures that constrain development and therefore occurrence of a given insect species (Wagner et al. 1984). This was explicitly accounted for by the Briere 1 model, which restricts development below 12.6° C. Other estimates of lower temperature threshold for degree-day models indicated similar values, ranging from 11.1 to 14° C (Liu and Tsai 2000; Nava et al. 2010). Oviposition of *D. citri* is another process that extreme temperatures affect. It has been demonstrated that *D. citri* does not oviposit below 16° C or above 41.6° C (Hall et al. 2011). These previous biological studies support the importance of considering extreme temperatures as factors influencing *D. citri* distribution.

Regarding performance of MaxEnt, the computed AUC values for both test and train data showed a good fit, given they exceeded the acceptable threshold of 0.7 (Phillips and Dudík 2008; Baldwin 2009). On the other hand, the sample size used in this work comprised 4,514 records for training and 1,934 for testing and, given that MaxEnt was considered among the best with predictive power across different sample sizes (Hernandez et al. 2006; Wisz et al. 2008), we expected the estimated habitat distribution to be representative of the insect's potential to occur in Mexico. This sample size was larger than the range of 10 to 30 point data, which is considered as too restrictive to make inferences on the species distribution (Wisz et al. 2008), and exceeds the 50-100 points estimated to obtain a useful distribution model (Wisz et al. 2008; Franklin and Miller 2009). On the other hand, the best predictor variables are the same for testing and for training the model, therefore indicating that they are both valuable to modeling as for predicting. In any case, the best predictor variables indicated that extreme temperatures affect *D. citri* distribution, which is supported by independent research (Yang et al. 2006; Hall et al. 2011).

### Development and population growth rate Distributions

The distributions of development and population growth rates were wider than for MaxEnt estimates. In the case of rm distribution, it was very similar to development, which probably results from the fact that as the development rate increases, the generation time decreases and rm increases (Poole 1974). The wider distribution of both variables as compared to habitat seems to occur because models based on underlying physiological or population responses, like development rate and rm, usually estimate the larger, potential niche, as compared to the smaller, realized niche estimated by point data (Buckley et al. 2010; Venette et al. 2010). Another probable factor affecting a wider distribution is that these indices were computed using a single predictor variable, while habitat was constrained by actual presence and by several bioclimatic layers. Both rates were dependent on annual mean temperature only, and additional environmental factors influence the inherent capacity to develop and grow; however, temperature is considered as the most important variable that affects insect development and has been considered as the primary factor to estimate species distribution when mechanistic models are used (Buckley et al. 2010). For example, in Australia, temperature was selected as the main factor affecting *D. citri* distribution under climate changes (Aurambout et al. 2009).

The close relationship among probability values and citrus host area was most likely because citrus plants are the preferred host of *D. citri* (Tsai and Liu 2000; Halbert and Manjunath 2004). In general, the three linear models suggest that habitat was related to citrus area, and states with the largest area planted with citrus had a high potential for *D. citri* to establish and grow; therefore, the risk of huanglongbing transmission might increase. The presence of huanglongbing in Mexico occurs in regions that, according to these results, can be considered as among the best suited for the vector to develop: Baja California Sur, Chiapas, Colima, Michoacan, Sinaloa, Hidalgo, Nayarit, Quintana Roo, San Luis Potosi, and Yucatan (SENASICA 2011). These results highlight the necessity to implement immediate measures aimed to detect, eliminate, confine, or control the huanglongbing inoculum sources. Important citrus producing areas (Figure 1) that have not been invaded by huanglongbing are the states of Veracruz, Tamaulipas, and Nuevo Leon. In such places, the need to establish a monitoring- alarm system for the rapid detection and actions against the disease is crucial.

Overall, the results indicated that coastal states and Yucatán and Baja California peninsulas are highly suitable for the occurrence, fast development, and population growth of *D. citri*. Therefore, these regions can be considered of high risk for huanglongbing transmission given the potential of the vector to establish and increase its populations. These results also suggest that eradication of *D. citri* would be difficult given the suitability for its development. As in other countries, measures to contain the spread of huanglongbing should be effected by the integration of different methods, for example, area-wide management of the vector (Qureshi and Stansly 2010), use of healthy plant material, and removal of infected plants (Bassanezi et al. 2010; Mayorga 2010; National Research Council 2010).

## Abbreviations

**1/d**,: development rate;
**AUC**,: area under the curve;
**rm**,: intrinsic rate of increase
